# Inflammatory myofibroblastic tumor of the thyroid gland

**DOI:** 10.3389/fendo.2023.1156117

**Published:** 2023-05-15

**Authors:** Yiyang Zhang, Jia Liu

**Affiliations:** Department of Thyroid Surgery, General Surgery Center, First Hospital of Jilin University, Changchun, China

**Keywords:** inflammatory myofibroblastic tumor (IMT), thyroid gland, spindle cell, rare tumor, ALK

## Abstract

Inflammatory myofibroblastic tumor (IMT) is a mesenchymal tumor with low incidence, which is extremely rare in the thyroid. At present, there is a lack of understanding regarding the etiology, pathogenesis, diagnosis and treatment of thyroid IMT. To improve the understanding of the disease, this article reviews the pathogenesis, clinical manifestations, pathology and immunohistochemistry, diagnosis, therapy and prognosis of thyroid IMT.

## Introduction

Inflammatory myofibroblastic tumor (IMT) is a peculiar neoplasm, which consists of spindle cells with myofibroblastic and fibroblastic morphology, with inflammatory permeation of plasma cells, eosinophils and/or lymphocytes, which rarely metastasizes (1). IMT is anatomically widespread and generally occurs in abdominal soft tissues, with abnormal sites including the pancreas, somatic soft tissues, central nervous system, and liver (2–4). Approximately 5% of the IMT cases are located in the head and neck (5, 6), and IMT in the thyroid is extremely rare. Previous studies on IMT often included inflammatory pseudotumor (IPT) and plasma cell granuloma (PCG), but this classification is not recommended in the latest World Health Organization (WHO) soft tissue tumor classification (1). According to the WHO classification, 8 cases of thyroid IMT have been reported to date (7–14) (**Table 1**). Most of the studies on thyroid IMT are in the form of case reports, with scattered research results, unclear research direction, lack of unified standard and consensus, and lack of in-depth understanding. There is an urgent need for systematic and further research on the etiology, pathogenesis, diagnosis and treatment. This article reviews the pathogenesis, clinical manifestations, pathology and immunohistochemistry, diagnosis, therapy and prognosis of thyroid IMT, and comprehensively summarizes the existing research in order to establish an in-depth understanding of the disease.

**Table 1:** List of formerly reported IMT cases.

No./ref	Age (years)/Gender	Clinical manifestation	Hashimoto’s thyroiditis	Immunohistochemistry (*ALK*, SMA, MSA and Vim)	Treatment	Follow-up	Ultrasonic manifestation
1 (13)	18/female	3 cm painless right thyroid mass	no	Positive: SMA, Vim Negative: *ALK-1*	Subtotal thyroidectomy	No recurrence 9 months after surgery	Hyperechoic nodule with numerous calcifications
2 (12)	50/female	0.6 cm right thyroid mass	no	Positive: SMA Negative: *ALK-1*	Total thyroidectomy	Doing well 1 year after surgery	Hypoechoic mass
3 (11)	61/male	Painless right thyroid mass with swelling	no	Positive: SMA, *ALK-1*	Total thyroidectomy	Doing well 1 year after surgery	Hypoechoic with cystic degeneration
4 (9)	64/female	3.8 cm left thyroid mass	no	Positive: Vim, SMA Negative: *ALK-1*	Left subtotal thyroidectomy + right partial thyroidectomy	No recurrence 4 months after surgery	Hypoechoic mass
5 (10)	57/male	Painless, 3 cm mass in the left and 4 cm in the right with hoarseness	no	Positive: ALK-1, SMA,Vim	Subtotal thyroidectomy + radiation therapy + steroid therapy	Alive with recurrence and relapse	Hypoechoic mass and heterogeneous echo
6 (14)	12/male	3.5 cm left thyroid mass	no	Positive: Vim, SMA Negative: *ALK*	Resection of the left lobe of thyroid, left banded muscle and part of sternocleidomastoid muscle + levothyroxine sodium replacement therapy	No recurrence during the 4-year follow-up	Heterogeneous hypoechoic region
7 (7)	66/male	8 cm right thyroid mass with hoarseness	no	Positive: Vim, SMA Negative: *ALK*	Right cervical lymphadenectomy (groups 2, 3, and 4) + right thyroidectomy + left subtotal thyroidectomy	No tumor recurrence or metastasis 6 months after surgery	Not provided
8 (8)	34/female	4 cm painless left thyroid mass	yes	Positive: *ALK-1*, SMA, Vim	Left lobectomy	Alive without recurrence 10 months after surgery	A hypoechoic mass

## Etiology and pathogenesis

The etiology and pathogenesis of IMT are unclear. It is considered that the development of IMT is mainly related to the following elements: inflammation, trauma, autoimmune diseases, surgery, viral infections and abnormal healing (mainly myofibroblast proliferation) (15, 16). In a recent study of 17 thyroid IMT cases, 9 of 17 patients were associated with inflammation, trauma, surgery and autoimmune etiology, which is consistent with the above inference (17). One of the 8 cases of IMT previously reported had goiter (9) and the focus showed inflammatory cell infiltration. The laboratory results revealed that retinol binding protein decreased and thyroglobulin (TG) significantly increased, suggesting that the cause may be associated with inflammatory infection or chronic inflammatory stimulation. Another patient suffered from Hashimoto’s thyroiditis (8), whose TG and anti-TG antibodies increased slightly, indicating that immune disorders and infection may be involved in the pathogenesis of IMT. However, there is still a lack of strong supporting evidence due to the limited number of cases. In addition, IMT is in connection with the rearrangement near the anaplastic lymphoma kinase gene (*ALK*) on chromosome 2p23 or in *ALK* (18). The *ALK* protein may be identified in about 50 to 60% of cases and has a good correlation with *ALK* gene rearrangement. *ALK*-negative IMT often shows other genetic abnormalities (7), including *TFG-ROS1, ETV6* or *ETV6-NTRK3*. In the 8 reported thyroid IMT cases, *ALK* was negative in 5 cases (7, 9, 12–14), but abnormalities of other fusion genes have not been reported.

## Clinical and imaging features

IMT patients are usually children and adolescents, with a female: male ratio of 2:1 (19). Approximately 5% of the cases were extraocular head and neck IMT (6). Unlike other sites, it has been reported in previous literature that thyroid IMT is predominant in women with an average age of 51 years (range 18-89 years) (14). However, in the 8 cases excluding PCG and IPT, thyroid IMT mainly occurred in those over 50 years old, and there was no gender difference. The clinical manifestations of IMT depend on the original location and the influence of the mass (20), and those of IMT in the head and neck are similar to inflammation (7). Thyroid IMT is not specific and may be asymptomatic. Most cases only show painless masses with slow progression in the thyroid area, and a few patients have uncomfortable symptoms of oppressing neighboring organs (such as the trachea and esophagus), including shortness of breath and dysphagia (14). If the patient has Hashimoto’s thyroiditis, symptoms associated with hypothyroidism may occur (17).

The imaging features of IMT are nonspecific, and both CT and MRI cannot distinguish between IMT and inflammation. Ultrasound is the best examination method, and is able to clarify the correlation between echoes, locations, boundaries, blood supply and lesions (17). In the reported thyroid IMT cases, most patients showed hypoechoic nodules, 1 case showed hyperechoic nodules, and 1 case showed a heterogeneous echo with microcalcifications, and it is suggested that it is difficult to distinguish IMT from thyroid carcinoma and infectious thyroiditis by ultrasonography.

## Pathology and immunohistochemistry

On the basis of the characteristics of myofibroblasts/fibroblasts, inflammatory cells and matrix, IMT can be classified into three basic histological morphologies (1): (I) mucous vascular type, consisting of a loose arrangement of full or slender myofibroblasts in edematous mucoid tissue, with ample blood vessels and a great number of plasma cells, eosinophils and lymphocytes infiltration, similar to granulation tissue or a reactive process; (II) compact spindle cell type, characterized by close fascicular spindle cell proliferation, accompanied by different mucus and collagen stroma and inflammatory infiltration, similar to various spindle neoplasms; (III) oligofibrous type, characterized by a transparent collagen matrix, low cell density of spindle cells and relatively sparse inflammatory infiltration, similar to scar or desmoid fibromatosis. One or more types are often seen in a single tumor. In the present study, most thyroid IMTs showed the second type, and a great number of spindle cells were observed to proliferate and closely arrange into bundles, accompanied by mature inflammatory cells with uniform distribution in the thyroid gland. It is also considered that thyroid IMT has different pathological types to IMT in other parts, most of which show infiltration into fibrous stroma by lymphocytes and plasma cells in varying degrees (11), and a few are mainly fibrous histiocytic hyperplasia (11–13). However, overall the study showed that the second type is dominant in thyroid IMT.

Immunohistochemical staining showed that IMTs demonstrate diffuse expression of vimentin (Vim), local or diffuse expression of muscle-specific actin (MSA) and smooth muscle actin (SMA), and expression of ALK, Actin, Desmin (Des) and Calponin (21, 22). Negative results included CD21, CD23, S100, CD34, CD117, myoglobin and Caldesmon. Positive expression of MSA, SMA, and Vim support the diagnosis of IMT (7). All reported thyroid IMTs were positive for SMA, basically positive for Vim, and no MSA negative cases were found, which was consistent with the above conclusion.

## Diagnosis and differential diagnosis

**Figure 1:**
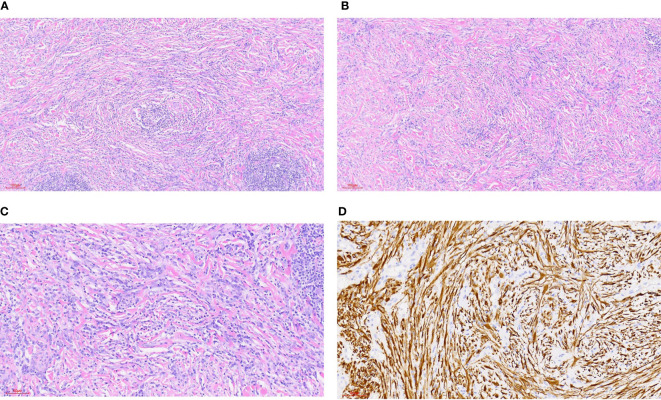
Inflammatory myofibroblastic tumor. **(A)** HE staining 100x (can also be marked as 10 ×): bundled myofibroblasts, interstitial infiltration of lymphocytes and plasma cells, and local lymphoid follicle formation. **(B)** HE staining 100x (can also be marked as 10 ×): bundled myofibroblasts with different degrees of collagenization between cells. There were more lymphocytes and plasma cells infiltration in the stroma. **(C)** HE staining 200x (can also be marked as 20 ×): bundled myofibroblasts with infiltration of lymphocytes and plasma cells in the stroma. **(D)** Immunohistochemical ALK1 staining 200x (can also be marked as 20 ×): positive.

**Figure 2:**
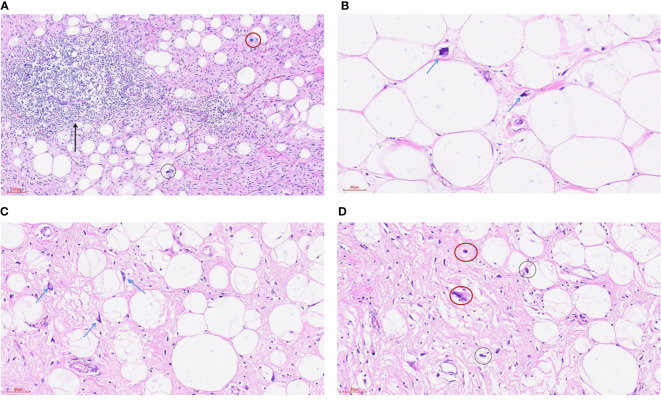
Highly differentiated liposarcoma-inflammatory subtype. **(A)** HE staining was 100x (can also be marked as 10 ×): the tumor was mainly composed of mature adipose tissue and a dense collagen fibrosis area. A small number of adipoblasts (black circle) were scattered in the mature adipose tissue, and scattered nuclear dark staining and irregular abnormal spindle cells (red circle) could be seen in the fibrous tissue. A large lymphocyte infiltration and the formation of local lymphoid follicles (arrowheads) were seen in the tumor. **(B)** HE staining 200x (can also be marked as 20 ×): mature adipose tissue and scattered adipoblasts (arrowhead). **(C)** HE staining 200x (can also be marked as 20 ×): mature adipose tissue and scattered adipoblasts (arrowhead). **(D)** HE staining 200x (can also be marked as 20 ×): the tumor is mainly composed of mature adipose tissue and a dense collagen fibrosis area, a small number of adipoblasts (black circle) are scattered in the mature adipose tissue, and scattered nuclear dark staining and irregular abnormal fusiform cells (red circle) can be seen in the fibrous tissue.

**Figure 3:**
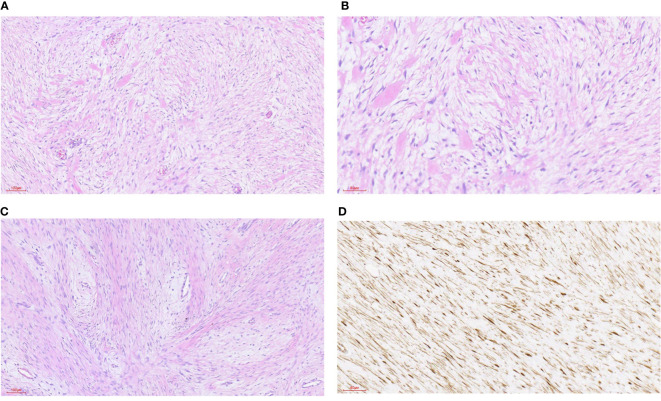
Desmoid fibromatosis. **(A)** HE staining was 100x (can also be marked as 10 ×): the tumor was composed of hyperplastic spindle fibroblasts and myofibroblasts as well as a small amount of collagen fibers. **(B)** HE staining 200x (can also be marked as 20 ×): the tumor consists of proliferated spindle fibroblasts and myofibroblasts as well as a small amount of collagen fibers. **(C)** HE staining was 100x (can also be marked as 10 ×): the tumor was composed of hyperplastic spindle fibroblasts and myofibroblasts as well as a small amount of collagen fibers. The spindle cells were arranged in an intertwined shape with local interstitial myxoid degeneration. **(D)** Immunohistochemical staining of β-catenin was 200-fold (can also be labeled as 20 ×): the tumor nucleus was diffusely positive.

**Figure 4:**
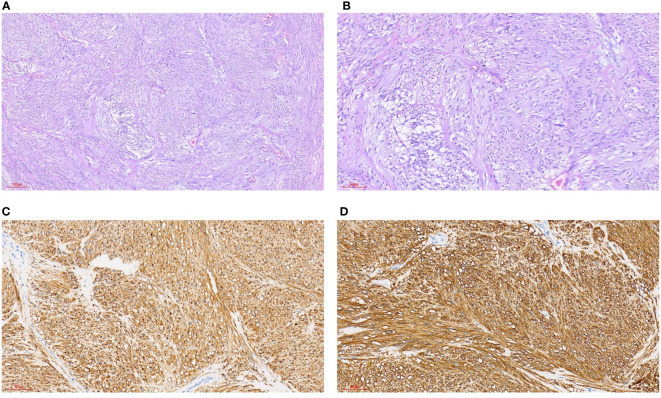
Gastrointestinal stromal tumors. **(A)** HE staining 100x (can also be marked as 10 ×): intertwined, bundle-like fusiform cells, focal interstitial mucinous degeneration. **(B)** HE staining 200x (can also be marked as 20 ×): interwoven, bundle-like fusiform cells, focal interstitial mucinous degeneration. **(C)** Immunohistochemical CD117 staining 200x (can also be marked as 20 ×): positive. **(D)** Immunohistochemical DOG1 staining 200x (can also be marked as 20 ×): positive.

**Figure 5:**
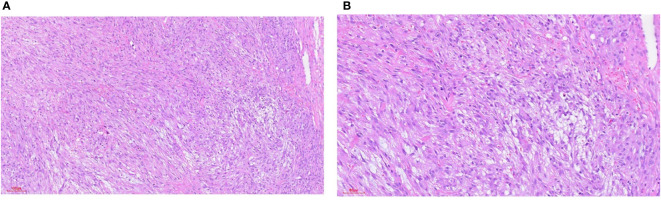
Nodular fasciitis. **(A)** HE staining 100x (can also be marked as 10 ×): intertwined fibroblasts/myofibroblasts, loose fissure-like background, containing a small amount of inflammatory cell infiltration, and interstitial infiltrating erythrocytes. **(B)** HE staining 200x (can also be marked as 20 ×): intertwined fibroblasts/myofibroblasts, loose fissure-like background, containing a small amount of inflammatory cell infiltration, and interstitial infiltrating erythrocytes.

IMT is diagnosed mainly by pathological and immunohistochemical detection. The essential diagnostic criteria proposed by the WHO are: “loose or compact fascicles of spindle cells with a prominent inflammatory infiltrate and a variable fibrous or myxoid stroma; expression of *ALK* (seen in as many as 60% of cases); and desirable diagnostic criteria are detection of *ALK* or other gene rearrangements” (1). Molecular tests for *ALK* can also be used for diagnosis, but this is generally not required (23). Pathological diagnosis may be challenging. In the European pediatric Soft Tissue Sarcoma Study Group (EpSSG) non-rhabdomyosarcoma soft tissue sarcoma (NRSTS) 2005 study, 80 patients were initially diagnosed with IMT; however, this changed in 20 patients after a national and international pathological review (24). In a phase II clinical trial of advanced IMT patients, 35 patients with locally diagnosed IMT were recruited, but only 24 patients were diagnosed with IMT (25). The misdiagnosed cases included various IMT-like entities (multifocal myofibromatosis, nodular fasciitis, calcified fibrous pseudotumor, fibrohamartoma, etc.). This study suggests that an accurate diagnosis following a second opinion by mesenchymal tumor pathologists may be required (26), or routine immunohistochemical tests may be performed to confirm (17). An important means of diagnosis and differential diagnosis is immunohistochemical staining, in which the positive results of SMA, MSA and Vim support the diagnosis of IMT (7) and can be used to distinguish IMT from other tumors originating from fibroblasts and smooth muscle cells. However, for patients who suffer from autoimmune diseases (such as Hashimoto’s thyroiditis), differential diagnosis should be made combined with clinical manifestations (27). The differential diagnosis of IMT (**Figure 1**) includes different non-neoplastic and neoplastic lesions showing spindle cell proliferation (28) (**Table 2**), such as inflammatory well-differentiated or dedifferentiated liposarcomas (**Figure 2**), inflammatory fibroid polyps, desmoid fibromatosis (**Figure 3**), gastrointestinal stromal tumors (GIST) (**Figure 4**), nodular fasciitis, IPT, and IgG4-related diseases (**Figure 5**).

**Table 2:** Differential diagnosis of inflammatory myofibroblastic tumor.

Types of diseases/Markers	S100	CD21	CD23	CD34	CD117
IMT	negative	negative	negative	negative	negative
Inflammatory pseudotumor		Almost all are negative (less than 5% of cases are positive)		Almost all are negative (less than 5% of cases are positive)	Almost all are negative (less than 5% of cases are positive)
Fibrous histiocytoma	Almost all are negative (less than 5% of cases are positive)	Occasionally positive (<15%, ≥5% positive cases)		Almost all are negative (less than 5% of cases are positive)	Almost all are negative (less than 5% of cases are positive)
Calcifying fibrous tumor	Usually positive (<95%, ≥75% positive cases)			Sometimes positive (<55%, ≥35% positive cases)	
Follicular dendritic cell tumor	Occasionally positive (<15%, ≥5% positive cases)	Usually positive (<95%, ≥75% positive cases)	Often positive (<75%, ≥55% positive cases)		Almost all are negative (less than 5% of cases are positive)
Interdigitating dendritic cell tumor	Almost all are positive (≥ 95% of cases are positive)	Almost all are negative (less than 5% of cases are positive)	Almost all are negative (less than 5% of cases are positive)		
Sarcomatoid carcinoma	Almost all are negative (less than 5% of cases are positive)	Almost all are negative (less than 5% of cases are positive)		Almost all are negative (less than 5% of cases are positive)	Occasionally positive (<15%, ≥5% positive cases)
Inflammatory leiomyosarcoma	Occasionally positive (<15%, ≥5% positive cases)	Almost all are negative (less than 5% of cases are positive)		Often positive (<75%, ≥55% positive cases)	Almost all are negative (less than 5% of cases are positive)
Desmoid fibromatosis	Almost all are negative (less than 5% of cases are positive)			Almost all are negative (less than 5% of cases are positive)	A small number of positive cases (<35%, ≥15%)
Gastrointestinal stromal tumors	Occasionally positive (<15%, ≥5% positive cases)			Often positive (<75%, ≥55% positive cases)	Usually positive (<95%, ≥75% positive cases)
Myxofibrosarcoma				Usually positive (<95%, ≥75% positive cases)	
Rhabdomyosarcoma	Occasionally positive (<15%, ≥5% positive cases)	Almost all are negative (less than 5% of cases are positive)		Almost all are negative (less than 5% of cases are positive)	Occasionally positive (<15%, ≥5% positive cases)
Malignant peripheral nerve sheath tumor	Often positive (<75%, ≥55% positive cases)			A small number of positive cases (<35%, ≥15%)	Almost all are negative (less than 5% of cases are positive)

## Treatment and prognosis

IMT is generally considered to be a borderline tumor, but thyroid IMT and extrathyroid IMT have different clinical behaviors (29). Thyroid IMTs are generally benign and rarely recur. The standard treatment for localized IMT is complete surgical resection (26). If resection is possible at the anatomical site, the main treatment is surgery. Patients who are not suitable for surgery are generally treated with combination therapy. The operative principle of IMT in the head and neck is that the important structures should be preserved as much as possible and the function should be given priority during radical resection (7). For patients who have incomplete resection or a positive resection margin, postoperative radiotherapy and chemotherapy are required (21). Thyroid IMT does not usually recur and metastasize. At present, thyroidectomy is the main treatment for thyroid IMT. With regard to the scope of thyroid IMT resection, it is suggested that the affected thyroid lobe should be removed, levothyroxine replacement and supplementary therapy should be given after surgery, and thyroid function should be tested (17).

Approximately 25% of extrapulmonary IMTs may recur, which depends to a certain extent on the anatomical location and the resectability of the tumor (2, 4, 19). Some scholars believe that ALK-negative IMT may metastasize, but the probability of recurrence does not seem to be related to ALK immunoreactivity (1). Another view is that the positive expression of ALK indicates a poor prognosis, and the overexpression of ALK is related to aggressive behavior (22). Research has shown that IMT of the thyroid gland is aggressive and can be attached to the surrounding structures (such as muscles, recurrent laryngeal nerve and esophagus) (10, 14), but most of the patients had a good prognosis, and only one patient relapsed with soft tissue metastasis 17 months after surgery (10). In a recent study (17), postoperative pathology showed no metastasis in patients who underwent intraoperative lymph node dissection, and imaging data (thyroid and abdominal ultrasound and lung CT scan) were followed up. So far, no recurrence or distant metastasis has been observed in those 17 patients. It was also pointed out in the literature that patients with high TG are more likely to relapse after a period of time following surgery (9), but there is still a lack of clinical evidence. Due to the good prognosis, priority should be given to preserve the functions of important structures (such as nerve, trachea or esophagus) during surgery, but clinical, biological and imaging follow-up is still crucial due to the limitations related the number of cases and long-term follow-up data.

## Conclusion

IMT of the thyroid is a rare tumor consisting of spindle myofibroblasts. Its etiology is unknown and may be related to inflammation and immune abnormalities. However, it is not clear whether there are fusion genes other than ALK and the effect of fusion genes in the pathogenesis. The clinical manifestations of the disease have no obvious specificity, and most of them are painless masses, which can be found by preoperative ultrasound. Pathological and immunohistochemical detection are the main means of diagnosis. Treatment of this disease mainly consists of surgery, and the postoperative prognosis is good, with rare recurrence and metastasis. At present, it is considered that this disease has a better prognosis compared with other IMTs, but there is still a lack of sufficient clinical evidence.

## Author contributions

YZ wrote the manuscript. JL provided critical revisions. All authors contributed to the article and approved the submitted version.
